# Amputation neuroma mimicking lymph node metastasis of remnant gastric cancer: a case report

**DOI:** 10.1186/s40792-017-0396-x

**Published:** 2017-12-12

**Authors:** Kenichiro Furukawa, Yutaka Tanizawa, Kimihide Kusafuka, Noriyuki Nishiwaki, Keiichi Fujiya, Hayato Omori, Sanae Kaji, Makoto Hikage, Rie Makuuchi, Tomoyuki Irino, Masanori Tokunaga, Etsuro Bando, Taiichi Kawamura, Masanori Terashima

**Affiliations:** 1 Division of Gastric Surgery, Shizuoka Cancer Center, 1007 Shimonagakubo, Nagaizumi-cho, Sunto-gun, Shizuoka 411-8777 Japan; 2 Division of Pathology, Shizuoka Cancer Center, 1007 Shimonagakubo, Nagaizumi-cho, Sunto-gun, Shizuoka 411-8777 Japan; 3 Division of Surgery, Sendai City Hospital, 1-1-1, Asuto-nagamachi, Taihaku-ku, Sendai-shi, Miyagi 982-8502 Japan; 4 Division of Gastric Surgery, National Cancer Center Hospital East, 6-5-1, Kashiwanoha, Kashiwa-shi, Chiba 277-8577 Japan

**Keywords:** Traumatic neuroma, Amputation neuroma, Remnant stomach, Gastrectomy, Gastric cancer, Lymph node metastasis

## Abstract

**Background:**

Amputation neuromas (ANs) are reactive hyperplasia of nerve tissues that occur after a trauma or surgery involving the peripheral nerves. Only two previous reports of ANs occurring around the stomach and post gastrectomy have been reported. We report the case of a patient with AN near the remnant stomach who underwent distal gastrectomy for gastric cancer.

**Case presentation:**

A 76-year-old man underwent distal gastrectomy, D1+ lymphadenectomy, and Billroth-I reconstruction for early gastric cancer in another hospital at 63 years of age. A regular gastrointestinal endoscopic follow-up examination after gastrectomy revealed an ulcerative lesion on the lesser curvature of the remnant stomach, which was diagnosed as remnant gastric cancer based on the histopathological examination. Then, he was transferred to our hospital. An upper gastrointestinal series and endoscopy revealed an 18-mm Type 0-IIc lesion on the lesser curvature of the remnant stomach with an estimated depth within the mucosa (T1a). An abdominal contrast-enhanced computed tomography (CT) failed to detect the primary lesion; however, a slightly enhanced 13 × 10-mm nodule was detected near the lesser curvature of the remnant stomach. An endoscopic ultrasonography-guided fine needle aspiration (EUS-FNA) of the nodule showed no cancer cell; thus, endoscopic submucosal dissection (ESD) for the remnant gastric cancer was performed. Histopathological examination revealed noncurative resection due to T1b2 and UL (+). We planned an additional surgical resection. Before the resection, CT was performed, which had a 3-month interval with a previous CT, showing an enlargement of the nodule to 16 × 12 mm. We diagnosed the nodule as a lymph node metastasis and performed resection of the remnant stomach, D2 lymphadenectomy, splenectomy, and Roux-en-Y reconstruction. The nodule was later diagnosed as AN based on the histopathological examination. There was no residual cancer in the resected specimen.

**Conclusions:**

We report AN mimicking lymph node metastasis near the remnant stomach of a patient with remnant gastric cancer. When nodules appear in the previous operative field, the possibility of ANs should be considered, although the incidence may be quite low.

## Background

Amputation neuromas (ANs) are reactive hyperplasia of nerve tissues that occur after a trauma or surgery involving the peripheral nerves [1, 2]. There are many ANs reported around the bile duct in the abdominal cavity [2–4]. However, to the best of our knowledge, only two previous reports of ANs occurring around the stomach and post gastrectomy have been reported [5, 6]. We report the case of a patient with AN near the remnant stomach who underwent distal gastrectomy for gastric cancer.

## Case presentation

**Fig. 1:**
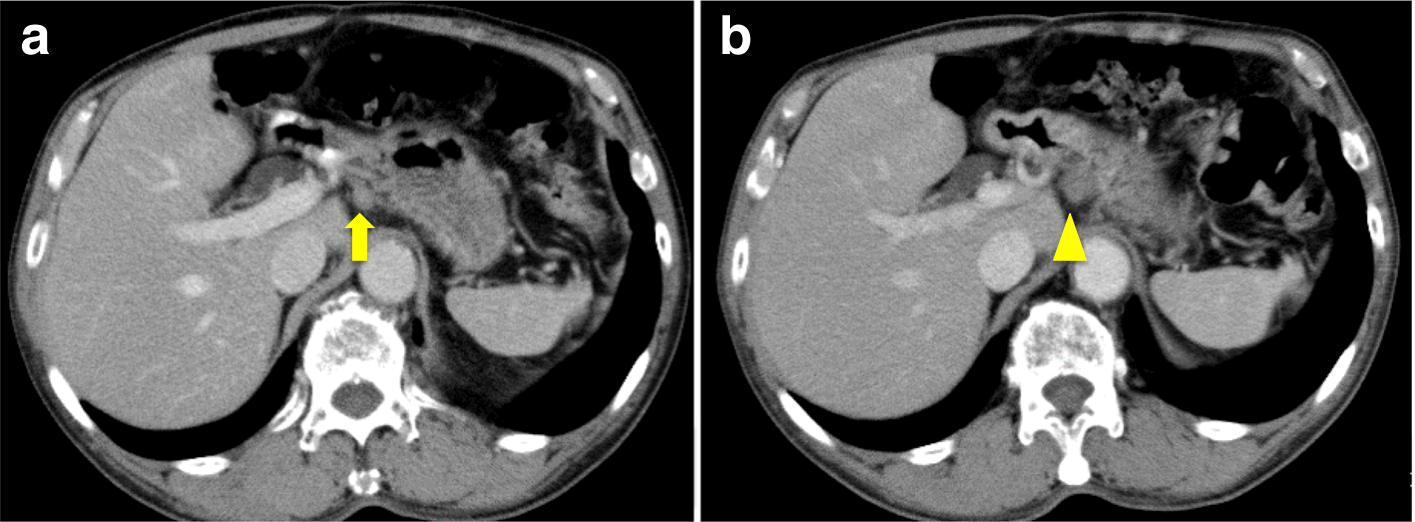
An abdominal contrast-enhanced computed tomography **a** before endoscopic submucosal dissection (ESD) and **b** 1.5 months after ESD. **a** Abdominal contrast-enhanced computed tomography shows a nodule lesion with slight contrast near the remnant stomach (arrow). **b** The nodule lesion increased in size over 3 months (arrow head)

**Fig. 2:**
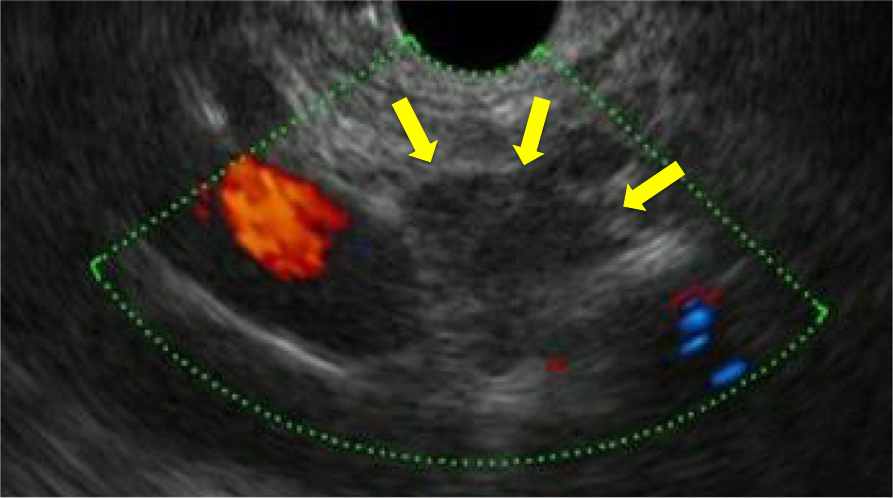
Endoscopic ultrasonography image of the nodule. An endoscopic ultrasonography shows a hypoechoic lesion without vascular flow. The margin of the lesion was slightly indistinct (arrows)

**Fig. 3:**
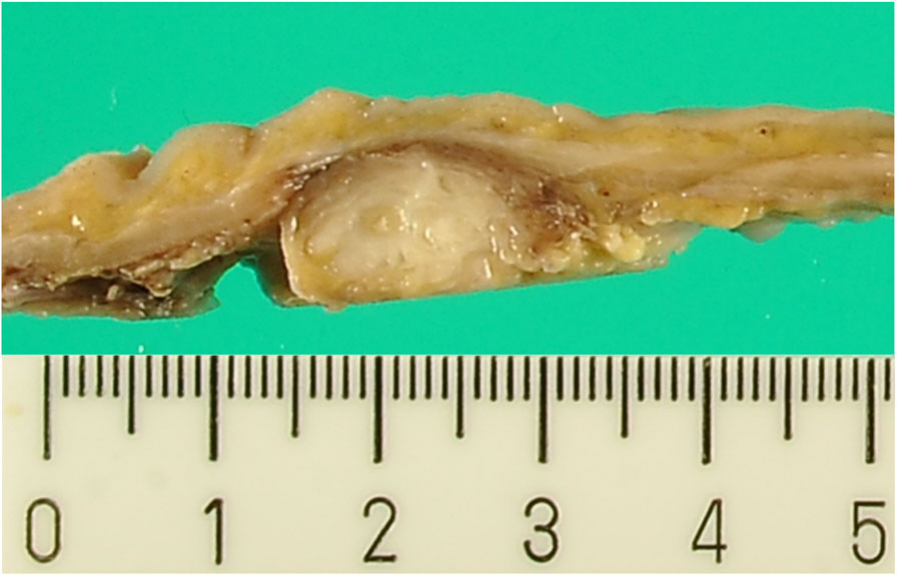
Macroscopic findings of the resected specimen. Macroscopically, the nodule suspected as lymph node metastasis was relatively well defined but did not have a clear capsule

**Fig. 4:**
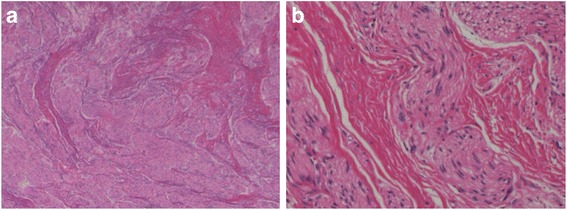
Histopathological findings. Thick nerve fibers without atypia proliferated with a bundle-like or spiral pattern. Hematoxylin–eosin staining. **a** × 20 and **b** × 100

A 76-year-old man who suffered from remnant gastric cancer was transferred to our hospital. He previously underwent distal gastrectomy, D1+ lymphadenectomy, and Billroth-I reconstruction for early gastric cancer in another hospital at 63 years of age. The histopathological examination showed pStage IA gastric cancer according to the Japanese Classification of Gastric Carcinoma, 14th edition [7]. A gastrointestinal endoscopic checkup after gastrectomy revealed an ulcerative lesion located on the lesser curvature of the remnant stomach. The histopathological examination of a biopsy specimen revealed well-differentiated tubular adenocarcinoma. After being transferred to our hospital, hematologic examinations including tumor markers, such as carcinoembryonic antigen, α-fetoprotein, carbohydrate antigen 19-9, and carbohydrate antigen 125, were within the normal range, except for the total protein level, which was slightly low (6.5 g/dL). An upper gastrointestinal series showed a 12-mm Type 0-IIc lesion on the lesser curvature of the remnant stomach with an estimated depth within the mucosa (T1a). A gastrointestinal endoscopy revealed an 18-mm Type 0-IIc lesion on the lesser curvature of the remnant stomach, which also had an estimated depth within the mucosa (T1a). Tumor biopsy revealed well-differentiated tubular adenocarcinoma. An abdominal contrast-enhanced computed tomography (CT) failed to detect the primary lesion; however, a slightly enhanced 13 × 10-mm nodule was observed near the lesser curvature of the remnant stomach (Fig. 1a). An endoscopic ultrasonography (EUS) revealed a hypoechoic lesion without vascular flow, and the margin of the lesion was slightly indistinct (Fig. 2). An endoscopic ultrasonography-guided fine needle aspiration (EUS-FNA) of the nodule showed no cancer cells. Following a clinical diagnosis of early gastric cancer confined to the mucosa (T1a) without lymph node metastasis, endoscopic submucosal dissection (ESD) was performed. The histopathological examination revealed Type 0-IIc, 25 × 15 mm, tub 1 > tub 2, T1b2 (SM 2, 625 μm), UL (+), ly (−), v (−), HM 0, VM 0. So, the ESD was diagnosed as noncurative according to the Japanese Gastric Cancer Treatment Guideline, 14th edition [8], and an additional surgical resection was planned. Before the resection, CT was performed again, which had a 3-month interval with a previous CT, showing an enlargement of the nodule to 16 × 12 mm (Fig. 1b). We diagnosed the nodule as a lymph node metastasis and his remnant gastric cancer as early remnant gastric cancer, M-12-S, post-Billroth-I reconstruction, U, Less, Type 0-IIc, 25 mm, tub 1 > tub 2, pT1b2, cN1, cM0, cStage IB. The differential diagnoses of the nodule were lymph node without metastasis, hamartoma, schwannoma, neurofibroma, desmoids, inflammatory fibrosarcoma, and inflammatory myofibroblastic tumor. He underwent resection of the remnant stomach, D2 lymphadenectomy, splenectomy, and Roux-en-Y reconstruction. The nodule rigidly adhered to the remnant stomach and appeared to be a lymph node metastasis in #3a. Macroscopically, the relatively well-demarcated, yellowish-white nodule was in the ventral side of the remnant stomach; however, the 11 × 6-mm nodule had no fibrous capsule (Fig. 3). In the histopathological examination, no residual cancer in the resected specimen was detected; the nodule showed wavy proliferation of nerve fibers without atypia nor mitosis, and a hyalinized stroma was seen between nerve bundles (Fig. 4). Therefore, we diagnosed the nodule as AN. We did not perform immunohistopathological examinations because we were able to distinguish hyperplasia from neoplasm in the HE staining. The histopathological diagnosis was early remnant gastric cancer, pT1b2, N0, M0, pStageIA. The postoperative course was uneventful, and there is no evidence of recurrence 37 months after surgery.

## Conclusions

ANs, also known as traumatic neuromas, occur after a trauma or surgery involving the peripheral nerves. ANs are reactive hyperplasia of nerve tissues, rather than true neoplasms, and usually occur at the proximal end of severed nerves [1, 2]. Theoretically, any nerve that is encased by Schwann cells and whose continuities have been disrupted may subsequently form ANs [2].

In this patient, AN occurred around the lesser curvature of the remnant stomach. It is considered that AN arose from the end of the nerve severed during a lymphadenectomy for gastric cancer 12 years ago.

It is well known that ANs generally arise around the bile duct in the abdominal cavity [2–4]. Previous reports have demonstrated 0.5, 0.28, and 0.23% AN incidences in orthotopic liver transplantation, cholecystectomy, and biliary tract surgery, respectively [9–11]. Because delicate nets of nerve fibers surround the common bile duct, symptoms such as abdominal pain, pyrexia, vomiting, and jaundice commonly occur [5, 12]. To the best of our knowledge, only two previous reports of ANs occurring around the stomach and post gastrectomy have been reported; one occurred from the suture line of the gastric remnant [5] and the other from around the celiac trunk [6].

Several imaging features of ANs have been reported. ANs are oval and have a small short/long axis ratio. They are generally hypoechoic, but in some patients, central hyperechoic areas are observed in the nodules [1, 13]. While most reports have shown that ANs have no internal vascularity [14–16], Sung et al. [13] have reported that some ANs have vascularity on color Doppler examination. In general, ^18^F-fluorodeoxyglucose-positron emission tomography (FDG-PET) reveals no uptake [6, 13]. In the present patient, contrast-enhanced CT showed a slightly enhanced nodule, and EUS showed a hypoechoic lesion without vascular flow and a slightly indistinct margin. FDG-PET was not performed for this patient. We have two reasons for not performing FDG-PET. First, we were convinced that the nodule was lymph node metastasis clinically. Second, we do not perform FDG-PET routinely because the diagnostic availability of FDG-PET is insufficient in patients with gastric cancer. Although the image findings of the present patient matched some of those of ANs, they were atypical. In addition, contrast-enhanced CT showed enlargement of the nodule, which led to the clinical diagnosis of the nodule as lymph node metastasis.

The reason why the nodule increased in size after ESD is unclear. In the histopathological examination, there was no sign of proliferation, hemorrhage, nor inflammation. Therefore, it is probably due to the measurement error of contrast-enhanced CT.

If the possibility of AN was considered as a preoperative differential diagnosis, intraoperative frozen section diagnosis would have been performed, excluding the diagnosis of lymph node metastasis secondary to remnant gastric cancer. Thus, the splenectomy could have been avoided. In this patient, we did not perform intraoperative pathological examination because we had confidence that the nodule was lymph node metastasis due to following reasons: First, the nodule increased in size in the repeated contrast-enhanced CT. Second, the nodule appeared to be lymph node metastasis macroscopically during the operation.

We report a patient with remnant gastric cancer having AN mimicking lymph node metastasis near the remnant stomach. When nodules appear in the previous operative field, the possibility of ANs should be considered, although the incidence may be quite low.

## Abbreviations

AN: Amputation neuroma
CT: Computed tomography
ESD: Endoscopic submucosal dissection
EUS: Endoscopic ultrasonography
EUS-FNA: Endoscopic ultrasonography-guided fine needle aspiration
FDG-PET: ^18^F-Fluorodeoxyglucose-positron emission tomography

## References

[1] Yabuuchi H, Kuroiwa T, Fukuya T, Tomita K, Hachitanda Y (2004). Traumatic neuroma and recurrent lymphadenopathy after neck dissection: comparison of radiologic features. Radiology.

[2] Stembridge VA (1951). Amputation neuroma following cholecystectomy. Ann Surg.

[3] Goto W, Kanazawa A, Tsukamoto T, Shimizu S, Yamashita Y, NIshiguchi Y (2015). A resected case of amputation neuroma of the bile duct diagnosed by intraoperative frozen section examination. J Jpn Surg Assoc.

[4] Ueno Y, Ikeda K, Maehara M, Sakaida N, Omura N, Kurokawa H (2008). Traumatic neuroma of the bile duct. Abdom Imaging.

[5] Tani Y, Sanji T, Midorikawa M, Handa Y, Morita S, Oono H (1995). A case of amputation neuroma occurring in the sutured site of the remaining stomach. Prog Dig Endosc.

[6] Kwon JH, Ryu SW, Kang YN (2007). Traumatic neuroma around the celiac trunk after gastrectomy mimicking a nodal metastasis: a case report. Korean J Radiol.

[7] Japanese Gastric Cancer Association. Japanese classification of gastric carcinoma: 3rd English edition. Gastric Cancer. 2011;14:101–12. 10.1007/s10120-011-0041-5 21573743

[8] Japanese Gastric Cancer Association. Japanese gastric cancer treatment guidelines 2014 (ver. 4). Gastric Cancer. 2017;20:1–19. 10.1007/s10120-016-0622-4 PMC5215069 27342689

[9] Navez J, Golse N, Bancel B, Rode A, Ducerf C, Mezoughi S (2016). Traumatic biliary neuroma after orthotopic liver transplantation: a possible cause of “unexplained” anastomotic biliary stricture. Clin Transpl.

[10] Furukawa M, Nakata T, Yamada R (1981). Tankan Dantan Shinkeisyu No Ichirei (a case of amputation neuroma of the bile duct). J Biliary Tract & Pancreas.

[11] Iwasa M, Nakamura K, Kitamura J (1988). Tanteki Go Heisokusei Oudan Wo Kitashita Dantann Shinkeisyu No Ichirei Honpou Houkokurei No Kentou (a case of amputation neuroma with obstructive jaundice following cholecystectomy—review of the Japanese literature). J Biliary Tract & Pancreas.

[12] Shafiroff BG, Hinton JW (1950). Surgical anatomy of the choledochal nerves. Arch Surg.

[13] Sung HS, Kim YS (2017). Ultrasonographic features of traumatic neuromas in breast cancer patients after mastectomy. Ultrasonography.

[14] AlSharif S, Ferre R, Omeroglu A, El Khoury M, Mesurolle B (2016). Imaging features associated with posttraumatic breast neuromas. AJR Am J Roentgenol.

[15] Kwak JY, Kim EK, Kim MJ, Son E (2009). Sonographic features of traumatic neuromas after neck dissection. J Clin Ultrasound.

[16] Ha EJ, Baek JH, Lee JH, Kim YJ, Kim JK, Kim TY (2012). Characteristic ultrasound feature of traumatic neuromas after neck dissection: direct continuity with the cervical plexus. Thyroid.

